# Validation of Age Estimation Using the Compositional Variation of Dental Hard Tissue: An X-ray Diffraction Analysis

**DOI:** 10.7759/cureus.65696

**Published:** 2024-07-29

**Authors:** Arora Annanya, Ramya Ramadoss, Sandhya Sundar, Suganya Panneer Selvam, Hemashree K

**Affiliations:** 1 Oral Pathology and Oral Biology, Saveetha Institute of Medical and Technical Sciences, Chennai, IND

**Keywords:** peak intesity, d values, jcpds, xrd, age estimation

## Abstract

Introduction

X-rays and X-ray diffraction (XRD) are two non-destructive techniques that determine a material's crystallographic structure, chemical composition, and physical properties. They can also be used to estimate a person's age when it is unknown, assess the need for orthodontic treatment, or predict the likelihood of tooth decay. This research aims to validate the accuracy of age estimation through X-ray diffraction analysis of tooth hard tissue with compositional changes.

Methodology

Four tooth samples were analyzed using the Pan Analytical XRD (Malvern Panalytical Ltd., UK) unique d8 family platform, which uses Cu Kα radiation (0.154 nm) and a 0.02° scan rate from 0 to 80°.

Results

The angle of incidence (ω) is established between the sample and the X-ray source. The angle of diffraction, 2θ, is established between the detector and the incident beam. The detector angle 2θ and the incident angle ω always equal half. Inter-atomic distance, or d-spacing (D = 10^-8 cm in Angstrom units), is measured. A greater crystal size or a greater degree of crystallinity may be indicated by a higher peak intensity, which translates to an increased amount of atoms in the crystal.

Conclusion

The study's findings suggest that XRD is a promising new technique for age determination, as it has an advanced and dynamic approach to finding the crystallographic characteristics of the provided sample.

## Introduction

Age estimation plays a key role in forensic science and is applied in many different legal and investigative contexts. These settings include criminal investigations, the identification of unidentified human remains, the assessment of criminal responsibility, and the evaluation of a person's eligibility for legal rights and benefits [1]. In forensic odontology, age estimation is particularly significant due to the durability and resilience of dental hard tissues, such as enamel, dentin, and cementum, which can provide valuable insights into an individual's age at the time of death or examination [2].

Traditionally, age estimation in forensic odontology has relied on morphological and developmental features of teeth, such as tooth eruption patterns, dental maturation stages, and the degree of wear and attrition [3]. While these methods have been helpful in many cases, they often have limitations, such as variability in developmental patterns among individuals and reliance on subjective visual assessments [4]. Therefore, there is a growing need for more objective, reliable, and scientifically validated techniques for age estimation in forensic odontology.

X-ray diffraction (XRD) analysis has emerged as a promising method for investigating the compositional variation of dental hard tissues and assessing their crystalline structure. This XRD is a non-destructive analytical technique that uses X-rays to determine materials' crystallographic arrangement, chemical composition, and physical properties [5]. Examining the diffraction patterns formed when X-rays interact with the crystalline structure of dental tissues allows XRD to provide important information about the molecular makeup and structural integrity of these tissues [6].

Utilizing XRD, forensic odontology determines age by analyzing changes in the hard tissues of the teeth over time brought on by a variety of factors [7]. XRD analysis can be used to identify and quantify changes in the crystalline structure, mineral density, and elemental composition of dental tissues [8].

Analysis of the compositional variation of dental hard tissues, especially enamel and dentin, using XRD has been demonstrated to be useful in age estimation in previous research [9]. Certain XRD measurements, such as peak intensities and crystallite sizes, have been linked to an individual's age, according to studies. It can be inferred from this that these measurements may serve as trustworthy markers of age-related alterations in dental tissues [6].

To validate the use of XRD analysis for age estimation in forensic odontology, more investigation is necessary. Validation studies are essential to assess XRD-based age estimation methods' accuracy, precision, reliability, and reproducibility across diverse populations and sample types. Furthermore, to evaluate the performance of XRD analysis in real-world forensic casework scenarios, comparative studies with established age estimation techniques, such as radiographic assessment and histological analysis, are necessary.

The purpose of this study is to investigate the compositional variation of dental hard tissues, such as cementum, dentin, and enamel, in order to validate the use of XRD analysis for age estimation in forensic odontology. Through a comprehensive analysis of XRD data obtained from a diverse sample population, this research seeks to establish robust correlations between XRD parameters and chronological age, ultimately contributing to developing reliable and scientifically validated age estimation methods in forensic odontology.

## Materials and methods

**Figure 1:**
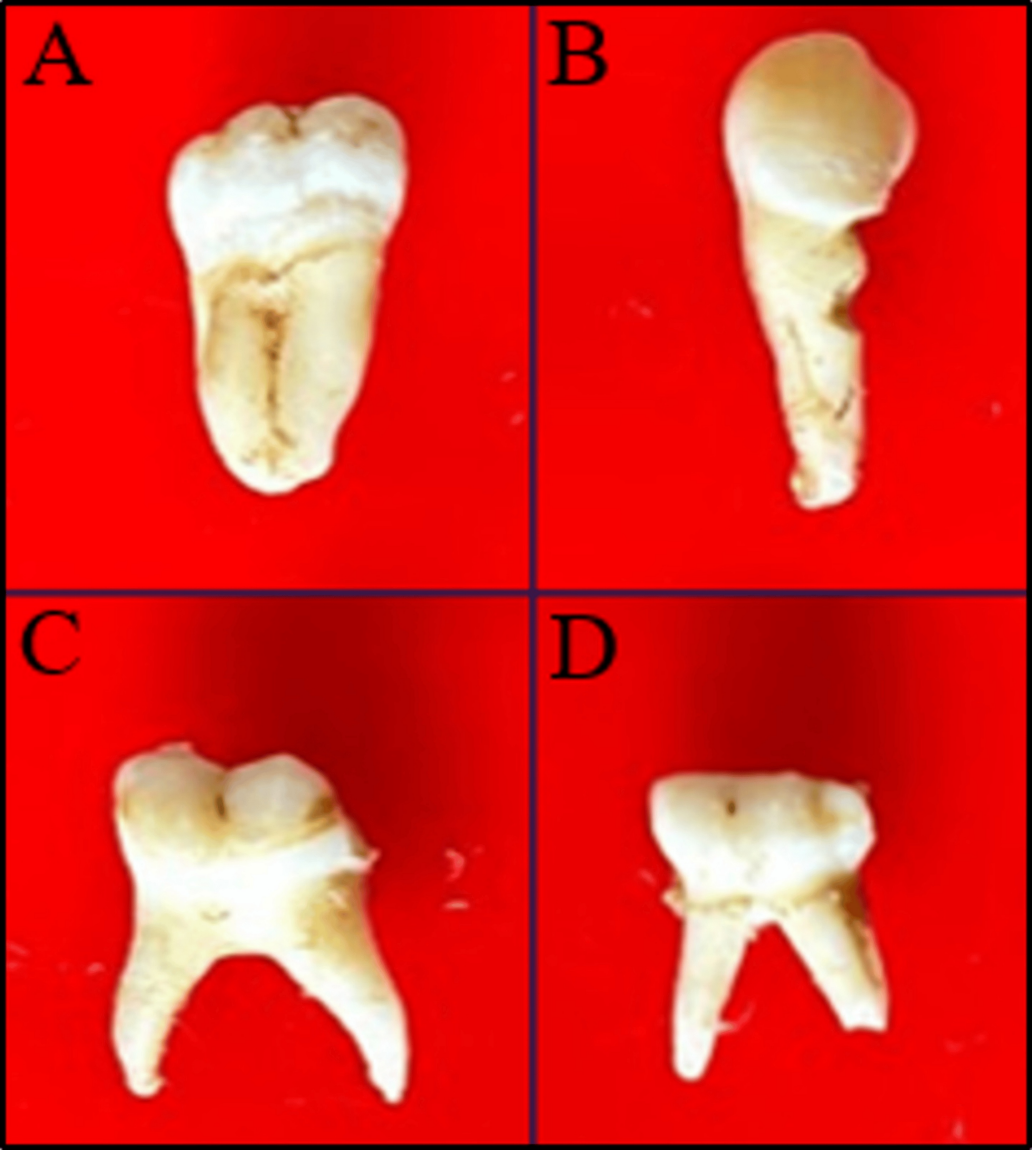
Teeth subjected to XRD analysis a: maxillary third molar; b: permanent mandibular canine; c: deciduous mandibular second molar; d: deciduous mandibular second molar XRD: X-ray diffraction

X-ray diffraction (XRD) analysis is a prompt and unambiguous method for investigating materials' crystalline structure and composition. The present study employed a Pan Analytical X-Ray Pro Diffractometer (Malvern Panalytical Ltd., UK) with Cu Kα radiation (0.154178 nm) and a 0.02° scan rate over 0-80° on a unique d8 family platform to perform XRD analysis of four tooth samples (Figures 1A-1D).

Several vital components were required to conduct XRD analysis of teeth, including tooth samples, an X-ray diffractometer, an X-ray source, a sample holder, a detector, radiation shielding, and data analysis software. The investigation utilized four tooth samples devoid of caries, attrition, and fractures. The samples needed to be homogeneous to ensure uniform results, even when only a tiny portion was analyzed from a bulk quantity. The study was conducted at Saveetha Institute of Medical and Technical Sciences, Chennai, India, and approved by the Institutional Review Board (SRB/SDC/OBIO-2226/24/119).

Before analysis, the tooth samples underwent thorough cleaning to eliminate dirt or pollutants and ensure they were dry (Figures 1A-1D). Calibration samples were subjected to appropriate temperatures using a NEY muffle furnace. The samples intended for X-ray experiments were then cut into 1 mm sheets using a diamond handpiece powder and transferred into respective plastic pouches.

Subsequently, the tooth samples were positioned on the imaging surface of the X-ray equipment, and machine settings such as exposure time and radiation intensity were adjusted based on the desired level of detail and the specific X-ray equipment used. The X-ray machine was then activated to capture images of the tooth samples, thereby revealing their interior structure, including dentin, enamel, pulp, and any potential dental disorders. The captured X-ray images were uploaded to a computer or digital system for editing, and specialized dental imaging software was employed to enhance and examine the images by adjusting contrast, brightness, and zoom settings for improved clarity. Finally, the results were obtained in the form of graphical representations.

## Results

**Figure 2:**
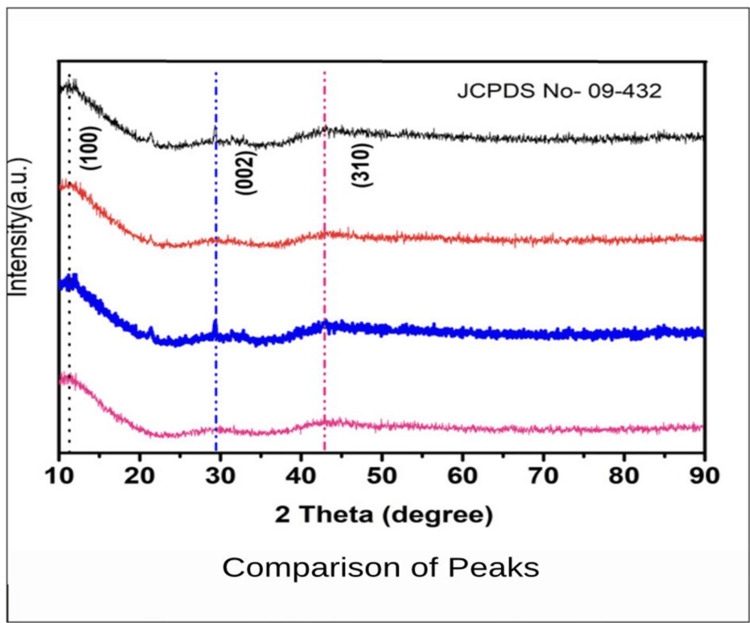
Comparison of absorption peaks across all four teeth

**Figure 3:**
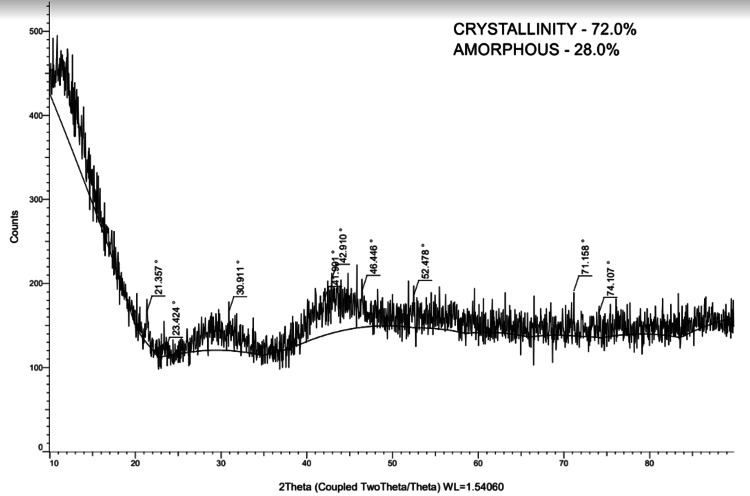
Absorption peak of maxillary third molar

**Figure 4:**
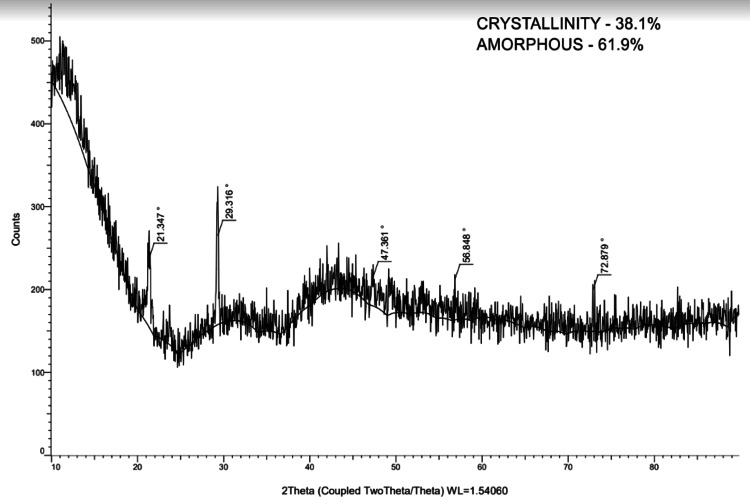
Absorption peak of permanent mandibular canine

**Figure 5:**
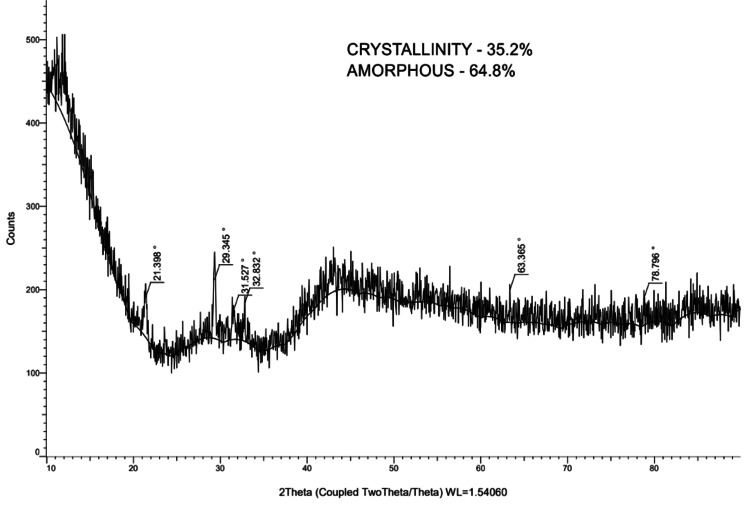
Absorption peak of deciduous mandibular second molar (1)

**Figure 6:**
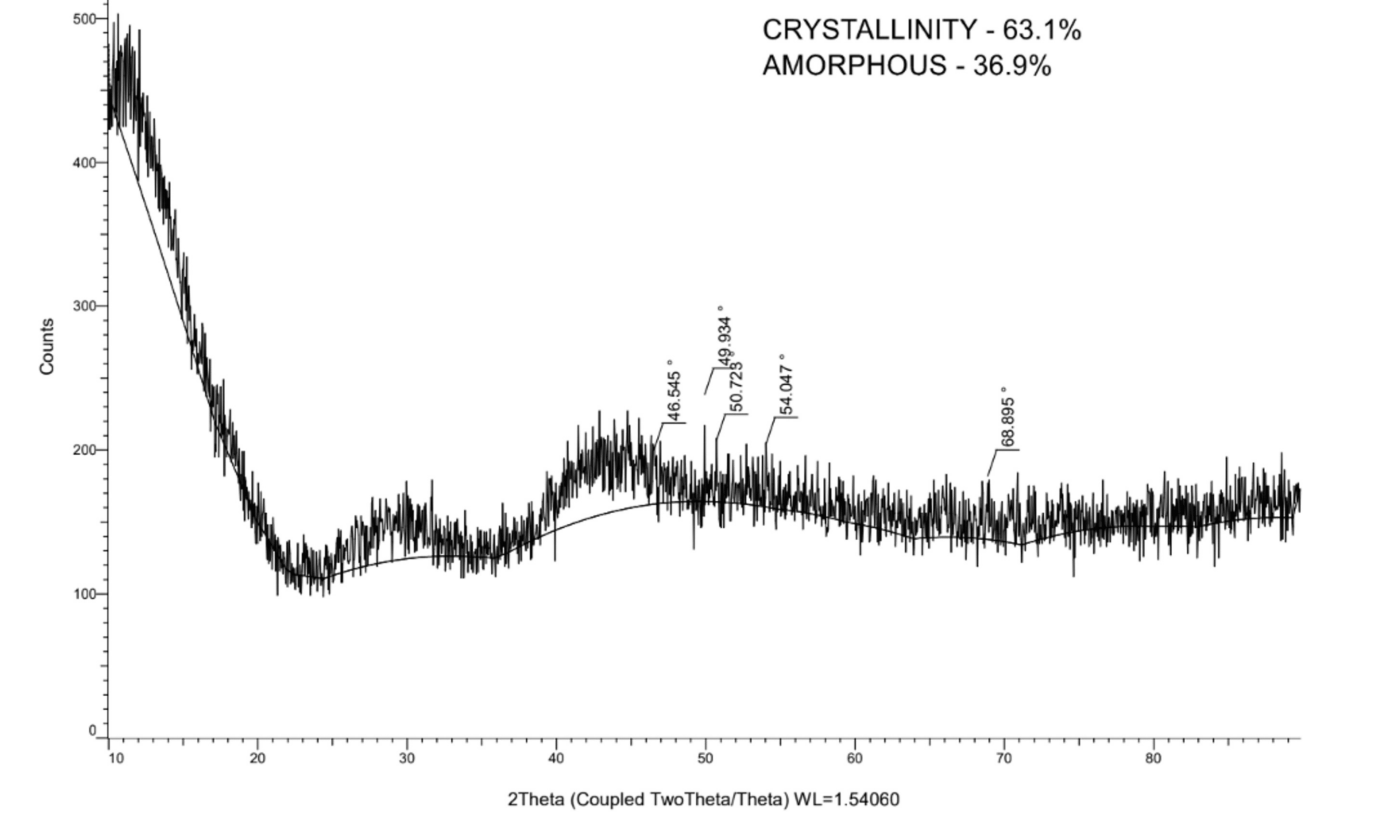
Absorbtion peaks of deciduous mandibular second molar (2)

In order to examine the structural characteristics of dental samples, the current study used X-ray diffraction (XRD) analysis (Figure 2) to analyze maxillary third molars (Figure 3), permanent mandibular canines (Figure 4), and deciduous second molars (Figures 5, 6). The diffraction angle (2θ), formed across the incident beams and the detector, and the incidence angle (ω), specified between the sources of X-rays and the sample, were the essential geometric relationships that underpinned the XRD process. It was important to note that ω was always equal to half of 2θ. 

Analyzing d-spacing, or the inter-atomic spacing, sheds light on important aspects of the characterization of the crystal lattices. Increased crystallinity or longer crystal sizes within the samples were correlated with the strength of XRD peaks, which indicated the quantity of atoms in the crystal.

The key characteristics of the peaks of teeth were compared (Table 1). XRD experiments were performed with a scanning velocity of 2 degrees/min at a 2θ angle ranging from 30 to 80 degrees. Using the XRD method, a crystalline solid's distinct characteristic pattern can be utilized as a "fingerprint" to identify it. The quantity of atom in the crystal that may scatter X-rays was correlated with the strength of an XRD peak.

**Table 1:** Angle, d-value, and net intensity of all four teeth

Parameter	Angle	d-value	Net intensity
Maxillary third molar	21.35	4.15	38.42
	23.42	3.79	4.39
	30.91	2.89	46.35
	41.99	2.14	40.53
	42.91	2.10	64.08
	46.44	1.95	43.79
	52.47	1.74	38.56
	71.15	1.32	53.52
	74.10	1.27	29.94
Permanent mandibular canine	21.43	4.15	83.18
	29.31	3.04	107.62
	47.36	1.91	35.48
	56.84	1.61	48.92
	72.87	1.29	50.12
Deciduous 2^nd^ molar (1)	46.54	1.94	38.07
	49.93	1.82	74.38
	50.73	1.79	42.57
	54.04	1.69	44.24
	68.89	1.36	43.91
Deciduous 2^nd^ molar (2)	21.39	4.14	47.42
	29.34	3.04	70.36
	31.52	2.83	34.68
	32.83	2.72	45.51
	63.36	1.46	39.03
	78.79	1.21	32.37

Particle size had an impact on the peaks, and the size of the crystallites was inversely related to their full width at half maximum (FWHM). Peaks needed to rise proportionately to maintain a constant peak area, as the peaked area (integral intensity) had to be preserved, and FWHM decreased as the crystallite size increased (Table 2). Consequently, it was often assumed that the distance among the parallel planes of atoms (FWHM), or the width of the diffraction peak in radians at a height halfway between the peak maximum and background, determined the exact location of the diffraction peaks.

**Table 2:** Gross intensity, relative intensity, and full width at half maximum (FWHM) of four teeth

Parameter	Gross intensity	Relative intensity	Full width at half maximum (FWHM)
Maxillary third molar	167.12	60.0%	0.119
	117.85	6.9%	0.279
	166.39	72.3%	0.439
	178.55	63.3%	0.377
	204.83	100.0%	0.685
	191.60	68.3%	0.504
	185.90	60.2%	0.485
	191.15	83.5%	0.226
	165.48	46.7%	0.325
Permanent mandibular canine	237.61	77.3%	0.212
	265.21	100.0%	0.153
	215.58	33.0%	0.415
	212.03	45.5%	0.429
	199.52	46.6%	0.250
Deciduous mandibular second molar (1)	200.42	51.2%	0.387
	238.62	100.0%	0.370
	206.53	57.2%	0.416
	204.53	59.5%	0.464
	181.70	59.0%	0.343
Deciduous mandibular second molar (2)	190.34	67.4%	0.265
	211.64	100.0%	0.214
	174.87	49.3%	0.565
	183.46	64.7%	0.416
	199.91	55.5%	0.411
	189.48	46.0%	0.349

## Discussion

Age estimation of human remains is crucial in forensic investigations and tragedies [10]. It helps identify individuals and bring closure to families [11]. Multiple techniques have been employed for age estimation, including X-ray diffraction (XRD), which has shown efficacy in providing micro-details of complex tissues [12]. Hiraishi et al. (2022) utilized XRD analysis on human third molars and bovine incisors and revealed significant differences in surface layers, microstrains, and crystal sizes between species [8]. This highlights the potential of XRD in distinguishing between human and animal remains, contributing to forensic investigations [8].

In another study, Limdiwala and Shah (2013) emphasized the advantages of XRD over conventional radiographs for sample interpretation [12]. It avoids inherent distortions in panoramic radiographs due to projection geometry, highlighting the reliability and ease of interpretation offered by XRD in dental analysis. Sgheiza et al. (2023) investigated the elemental composition and crystallite size variations in unburned dental tissues, revealing significant correlations with age and sex [13]. The study demonstrated the potential of XRD in uncovering age and sex-related differences in dental tissues, further corroborating its utility in forensic age estimation.

In our study, XRD analysis revealed distinct diffraction patterns for various dental samples, indicating differences in crystallographic structure. Critical parameters such as d-values and net intensity were obtained, providing valuable insights into the composition and characteristics of the teeth studied.

However, age estimation remains a multifaceted challenge, with various methods exhibiting distinct merits and limitations [14]. Kvaal's method, based on specific identity proofs, showed discrepancies between estimated and actual age, highlighting the need for further refinement [15]. Similarly, methods based on tooth cementum thickness lacked precision in estimating age in older adults [14]. Applying principal component regression (PCR) models based on Raman spectra showed promise but required separate validation for male and female donors due to significant sex-related differences [16]. Additionally, techniques like radiocarbon dating and racemization studies provided precise age estimates but relied on historical events and specific contextual factors [10].

Forensic anthropological techniques also showed potential in estimating the age of unidentified remains [11]. However, the elemental composition of teeth alone may not suffice for accurate age estimation without additional morphological or molecular analyses [8].

Overall, the findings underscore the complexity of age estimation and the need for comprehensive approaches combining multiple techniques for enhanced accuracy and reliability. XRD emerges as a valuable tool, offering detailed insights into the compositional variations of dental tissues and contributing to advancements in forensic science and dental research. The distinct "fingerprint" patterns observed in XRD peaks further emphasize its potential for identification purposes, paving the way for future studies and applications in forensic investigations.

Limitations of the study

This study, despite highlighting the potential of XRD in forensic age estimation, has limitations. The small sample size and focus on dental tissues may limit generalizability to other human remains. The reliance on specific XRD parameters requires further validation across diverse demographic groups. Additionally, post-mortem changes affecting XRD patterns were not considered, which could impact accuracy. Integration with other techniques may be necessary for more comprehensive and reliable age estimation.

## Conclusions

In conclusion, X-ray diffraction (XRD) analysis is a promising tool for age estimation in forensic investigations, particularly in dental analysis. The studies reviewed demonstrate the potential of XRD to provide detailed and precise age estimates by analyzing the compositional variation of tooth-hard tissues. Despite challenges and limitations associated with other age estimation methods, XRD offers distinct advantages, including its non-destructive nature and ability to capture micro-details of dental structures. The findings underscore the complexity of crystallographic analyses and highlight the need for further research and refinement in forensic age estimation techniques. Moving forward, integrating XRD with complementary methods and advancing data analysis approaches can enhance the accuracy and reliability of age estimation in forensic contexts, ultimately contributing to the resolution of criminal cases and providing closure to victims' families.
